# Affordable gait analysis using augmented reality markers

**DOI:** 10.1371/journal.pone.0212319

**Published:** 2019-02-14

**Authors:** Gergely Nagymáté, Rita M. Kiss

**Affiliations:** Department of Mechatronics, Optics and Mechanical Engineering Informatics, Budapest University of Technology and Economics, Budapest, Hungary; Toronto Rehabilitation Institute—UHN, CANADA

## Abstract

A typical optical based gait analysis laboratory uses expensive stereophotogrammetric motion capture systems. The study aims to propose and validate an affordable gait analysis method using augmented reality (AR) markers with a single action camera. Image processing software calculates the position and orientation of the AR markers. Anatomical landmark calibration is applied on the subject to calibrate each of the anatomical points with respect to their corresponding AR markers. This way, anatomical points are tracked through AR markers using homogeneous coordinate transformations, and the further processing of gait analysis is identical with conventional solutions. The proposed system was validated on nine participants of varying age using a conventional motion capture system on simultaneously measured treadmill gait trials on 2, 3 and 4.5 km/h walking speeds. Coordinates of the virtual anatomical points were compared using the Bland-Altman analysis. Spatial-temporal gait parameters (step length, stride length, walking base, cadence, pelvis range of motion) and angular gait parameters (range of motion of knee, hip and pelvis angles) were compared between measurement systems by RMS error and Bland-Altman analysis. The proposed method shows some differences in the raw coordinates of virtually tracked anatomical landmarks and gait parameters compared to the reference system. RMS errors of spatial parameters were below 23 mm, while the angular range of motion RMS errors varies from 2.55° to 6.73°. Some of these differences (e.g. knee angle range of motion) is comparable to previously reported differences between commercial motion capture systems and gait variability. The proposed method can be a very cheap gait analysis solution, but precision is not guaranteed for every aspect of gait analysis using the currently exemplified implementation of the AR marker tracking approach.

## 1. Introduction

Gait analysis is the instrumented systematic study of human motion for measuring body kinematics and dynamics, and is used in medicine and biomechanical research to assess and treat individuals with impaired walking capabilities [1] or to improve sports performance [2]. A typical optical based motion capture gait laboratory has several cameras placed around a walkway. The subject has markers located at anatomical landmarks or rigid groups of markers are applied to the body segments [3]. In the latter case, the anatomical points are calibrated as virtual markers in the coordinate systems of the rigid marker clusters [3]. Trajectories of the markers or the position and orientation of the rigid bodies are calculated from the several camera pictures by the system using stereophotogrammetry [3]. The motions of the underlying bones are estimated to yield the joint kinematics. These motion capture camera systems are expensive, therefore there is a constant demand for more affordable gait analysis solutions with similar accuracy.

One of the current trends in gait analysis is the use of low-cost motion sensors based on inertial measurement units (IMUs) which combine sensor data from accelerometers and gyroscopes. These sensors are attached to body segments and measure the orientation of the segments. High precision orientation estimation of the IMU modules are possible due to advanced sensor fusion and filtering. Often a constrained biomechanical model is used to estimate body kinematics from sensor orientation data [4]. Using properly tuned constrained models and precise orientation tracking of IMU sensors gait analysis or other predefined motion types can be reliably measured [5,6]. Whereas, if the constrained model is not accurate the measurement results can be biased, e.g. a commercial IMU based motion analysis system proved to be reliable on adults [6], but shows significant bias on children as calculates the model with adult leg lengths [7]. While these systems are affordable and mobile, they have limitations. Direct position tracking of the sensors is only possible by continuous or periodic integral drift corrections or zero speed update [8,9] as accelerometer sensor readings contain noise which is exponentially accumulated in the integrated position data. An example for zero speed update is to zero out the estimated velocity when the foot is predicted to be on the ground during a gait trial [8]. To overcome the integral error, another common solution is the regression of the position data to zero, thus eliminating the error due to integrated errors of sensor drift [8]. Consequently, inertial systems work well on periodic motions but are less suitable for the absolute position tracking of objects, and the joint kinematics of a motion analysis highly depends on the constrained biomechanical model.

There are initiatives where open source solutions are provided to replicate the stereophotogrammetry based functionality of motion capture systems with consumer grade cameras. Jackson et al. [10] offers a complex solution for necessary camera calibration and the synchronization of video inputs from multiple cameras. This approach is based on stereophotogrammetry, where the identifiable points of the tracked object have to be seen from different angles by multiple cameras. Another image processing approach is homography, which relates the transformation between two planes [11]. This is used in photography for panorama picture stitching or perspective correction and is also used in augmented reality (AR) to estimate camera pose from coplanar points and vice versa. It can identify rotations and translations (3D kinematics) of an AR marker relative to the camera focus point and the image plane by how the corners of the known geometry marker appear on the recorded image. Compared to continuously drifted or zero corrected IMU-s, the 6 degree of freedom tracking of AR markers make them possible to track the absolute position of external objects [12] and body segments if attached to them. Compared to stereophotogrammetry based alternatives [10], AR marker based tracking can work with one camera, although in this case the movement direction can be limited (e.g. treadmill walking).

AR was mostly mentioned so far in motion studies as a part of therapies [13], but not for the purpose of biomechanical motion tracking. Ortega-Palacios et al. describe a gait analysis system with augmented reality, but the localization of infra-red LED (light emitting diode) markers is still processed by stereophotogrammetry [14]. Sementille et al. used actual augmented reality markers to track the position of joints on a very simplified anatomical model [15]. None of the above research works validated the data acquired using a conventional motion analysis system.

The first aim of this research is to present a novel approach for gait analysis with a single commercial action camera using augmented reality markers based on the approach of tracking body segments by marker rigid bodies [3]. Therefore, no simplification of the anatomical model is required, a full six degree of freedom kinematic analysis of each body segment and joint is possible using conventional or open-source motion analysis solutions such as OpenSim (NIH Center for Biomedical Computation, Stanford University, http://opensim.stanford.edu/).

The second aim of the paper is to validate a possible implementation of the proposed approach by simultaneous measurements with a conventional motion capture system on treadmill gait trials of healthy subjects of varying age at different walking speeds, followed by comparing the coordinates of the tracked virtual anatomical points and calculations for comparing angular and spatial gait parameters.

## 2. Methods

This section firstly describes the technical details of the proposed system. Secondly, the validation method of the system is described, which compares the accuracy of the AR marker system to a conventional optical motion capture system.

### 2.1. Description of the proposed augmented reality based motion capture system

#### 2.1.1. Experimental procedure with the proposed system

The measurement protocol with the exemplifying implemented AR marker system has been registered and openly accessible in an online protocol description [16] with further illustrations. Gait trial with the present system starts by fixing specified AR markers onto the corresponding body segments of the subject using wide elastic bands to minimize soft tissue artifact [17]. All the markers have to be visible from the same direction during the complete trial, from where the camera is set up. The camera was set up about 1.5 meters behind the subject in order that each marker is visible on the camera in the whole movement range of the subject. In the present experiment the coordinate system was camera centered, so only the direct inaccuracies of the markers can be measured. The coordinate system could be arbitrary using another AR marker seen by the camera which defines the coordinate system position and orientation. In this solution, the exact orientation and position of the camera is irrelevant as long as each marker is well visible, thus the camera could also be a handheld smartphone. The drawback of this approach would be that the position and orientation detection error of the markers relative to the reference marker becomes multiplied compared to camera centered solution.

**Fig 1:**
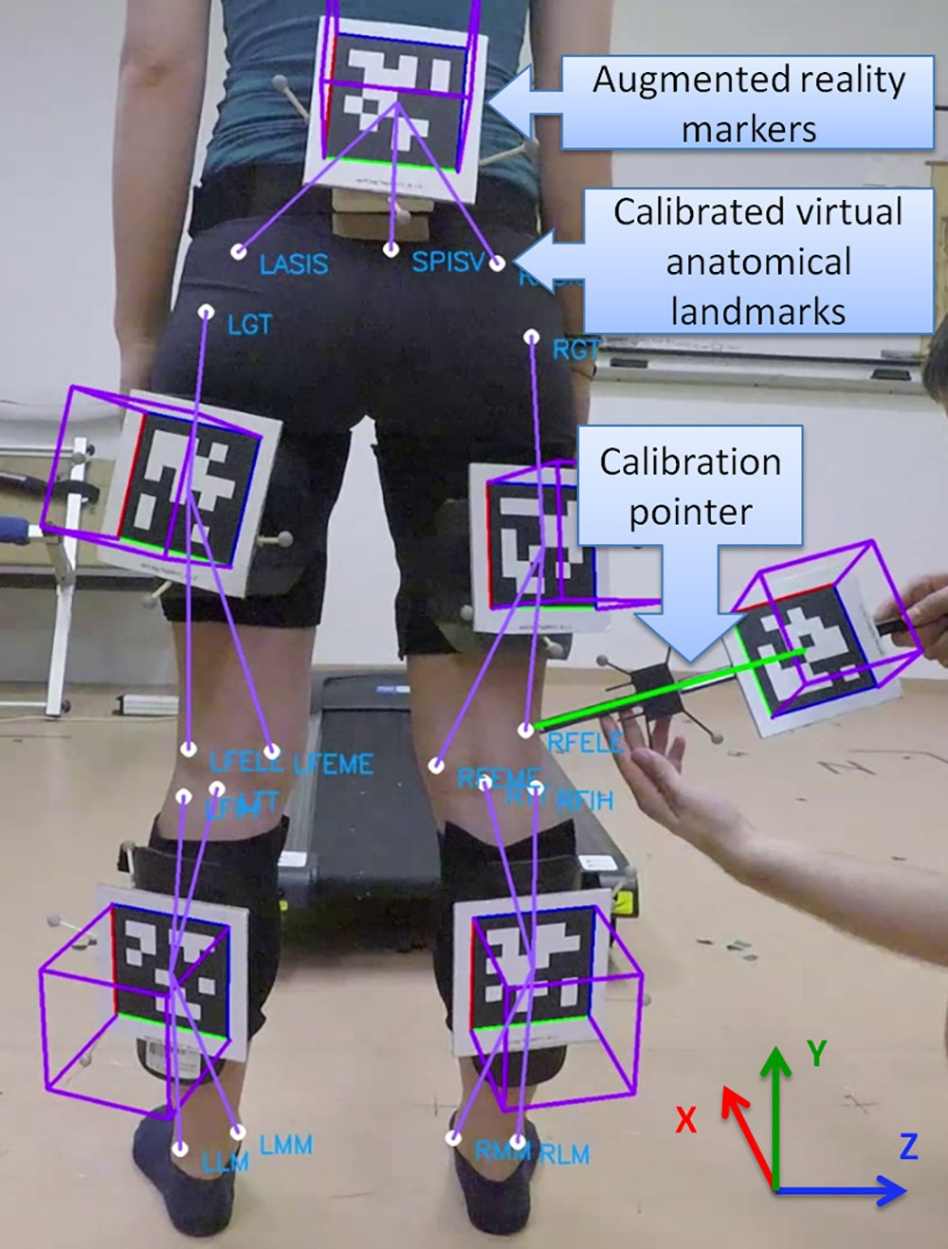
Calibration of anatomical points using the calibration pointer. The coordinate system illustrates the directions of the axes: axis *x* points in the forward direction of the movement, *y* points upward and *z* points to the right; however, the actual origin of the coordinate system is in the focus point of the camera. Purple squares are drawn on the markers by the processing software to display the proper orientation tracking of the markers. The white dots and labels of the anatomical points are also drawn by the software.

Before the measurement, anatomical landmark calibration has to be performed by palpation and with the help of a calibration pointer equipped with another AR marker (Fig 1). This procedure “teaches” the system the location of the indirectly tracked anatomical landmarks relative to their corresponding—directly tracked—AR markers using homogeneous coordinate transformation. Anatomical calibration is recorded by the camera and care must be taken so that the marker of the pointing wand and the calibrated body segment are well visible by the camera. Each anatomical point specified by the marker set has to be pointed with the pointing wand on the video. The calibration process takes about 1 to 2 minutes. Calibration is followed by gait trial on a treadmill for the desired time. The calibration and gait trial video files are processed offline by the image processing software where the frames of pointing to anatomical landmarks are selected manually. After this manual post-processing, a file with the calculated marker trajectories during the trial is available in a standard.trc file format. In the present experiment, the file is opened by a custom Matlab script which can perform calculations on marker trajectories and invokes a third party open-source biomechanical analyzing software (OpenSim) to calculate angular gait parameters.

The above procedure details the tested proof of concept implementation that we used. Further work should be invested in the optimization of the procedure where anatomical calibration and measurement evaluation is real-time (no post-processing), and the whole measurement could be performed even on a smartphone with a high-resolution camera and sufficient processing power.

#### 2.1.2. Acquisition system

The accuracy of AR marker pose estimation depends mainly on the quality of camera calibration, which eliminates optical distortions and sets the resolution of input images and marker size on the image in pixels. Therefore camera calibration is an important technical aspect of image processing and system accuracy, but does not form part of the conducted measurements. Camera calibration needs to be done only once when configuring system parameters. During the measurements there is no need to deal with camera calibrations. For the AR marker detection algorithm, a high shutter speed is important to avoid unrecognizable blurry images at faster motions. From the viewpoint of gait analysis, the highest possible frame rate (fps) is required for high temporal resolution. It is also essential for the camera to have a fixed zoom and fixed focal length (disabled autofocus) because camera calibration is valid for fixed values of these parameters.

Several cameras have been tested and calibrated with different settings (Table 1), but only the results with the setup that proved to be the optimal in terms of the above requirements are described in the paper, which is a GoPro Hero5 Black action camera (GoPro, Inc, San Mateo, California, USA) set to 2.7k resolution at 50 fps with 1/200 shutter speed in linear mode. The linear mode of this camera runs a factory calibrated image undistortion on the device and the recording will be free from optical distortions; only focal length is the information required from camera calibration performed in this mode.

**Table 1:** Tested cameras and calibrated camera parameters.

Camera	Resolution*	Frame rate (fps)	Shutter speed	Focal length (in pixels)	Distortion parameters [18]**
Kinect v2 (color video recording)	FullHD (1920x1080 pixel)	30	cannot be set	1034.68	k_1_: 0.0312 k_2_: -0.0450 k_3_: 0.0049
GoPro Hero 4 Silver	FullHD (1920x1080 pixel), narrow mode	60	cannot be set	1641.94	k_1_: -0.2971 k_2_: 0.1752 k_3_: -0.0755
GoPro Hero 5 Black	2.7k (2716x1524), linear mode	50	1/200	1483.71	k_1_: 0 k_2_: 0 k_3_: 0
GoPro Hero 5 Black	4k (3840x2160), wide mode	25	1/100	1775.89	k_1_: -0.2534 k_2_: 0.0894 k_3_: -0.0167

*Modes in GoPro cameras refer to the field of view option of the device; Kinect v2 has only a fixed wide field of view

**p_1_ and p_2_ distortion parameters are equal to 0 in each setup.

#### 2.1.3. Image processing

Camera calibration was performed by OpenCV using a chessboard pattern [18]. The processed video frames are undistorted with the OpenCV undistort function before the tag detection algorithm is called (it has no effect when the linear video mode is used with the GoPro).

AR marker detection and identification are performed by the Apriltag algorithm using the 36h7 marker tag family [19]. Position of the detected tags and the rotation matrix with respect to the camera are given by the Apriltag algorithm using homography trasformation which is available online (https://april.eecs.umich.edu/software/apriltag.html). The theory as well as the validation of the homography based orientation and pose estimation of the Apriltag algorithm can be found in the original paper of Olson [19]. In their validation on generated ground truth images, the angular error of the markers is less than 0.5° until about 75°off axis angle. The achievable accuracy of marker tracking in their results is significantly higher than with the more widespread ARtoolkit framework [19,20], which they graphically present [19]. Another experimental validation of Apriltag’s marker tracking accuracy has been conducted by Pfleging et al. with a motion capture system [12]. They found 4.3 (3.2) mm position error and 1.83° (1.77°) orientation error for a 58 mm side length Apriltag marker in a 0.8–1.2 m distance at 1280×720 camera resolution. Given the identity, position and orientation of the AR markers, the coordinates of the virtual anatomical points are calculated for each frame using homogeneous coordinate transformations as described in [21] and demonstrated with source code in S5 File. In the moment of calibration, the end point coordinates of the calibration pointer are taken as the coordinates of the calibrated anatomical point in the local coordinate system of the corresponding body segment determined by the AR marker (Fig 1).

### 2.2. Validation of the system

#### 2.2.1. Subjects

Ten subjects of varying age (ranging from 18–84 years) participated in the study (age: 28.6 (19.6) years, height: 1.71 (0.06) m, weight: 66.8 (17.8) kg). All participants were free from any musculoskeletal disorders. A written consent was given by the subjects after all necessary information about the procedure was presented. The study was approved by the National Science and Research Ethics Committee (21/2015).

#### 2.2.2. Reference system

An 18 camera OptiTrack Flex13 motion capture camera system (Natural Point, OR, USA) was used to simultaneously track the AR markers at matching sampling frequency with the video recording set to 50 Hz. Three infra reflexive motion capture markers were fixed on each AR marker defining a trackable rigid body in the Motive software (version: 1.10.2, Natural Point, OR, USA). Coordinate systems of the rigid bodies in Motive were aligned with the AR marker coordinate systems as it is identified by the Apriltag algorithm. This enabled the use of the same anatomical calibration on AR marker position and orientation, as well as the performance of the whole data processing described above on the same motion recorded by the two different systems. The only source of the deviations in the final gait parameters calculated by both systems is the tracking inaccuracies of the proposed solution that wanted to be identified, and possible inaccuracies of the action camera placement if the optical axis of the camera and the axis *x* of the motion capture system are not completely parallel. This latter error may only influence spatial gait parameters when only designated projections of anatomical points are used in the calculation (e.g. only the *x* coordinate of foot markers is used to calculate step size). The same applies to conventional motion capture systems when the patient’s trajectory or the placement of the treadmill is not completely parallel to the motion capture reference frame. This error is neglected in the comparison of the gait parameters but addressed in the anatomical point accuracy comparison.

#### 2.2.3. Measurement procedure

Every subject performed normal walking on a treadmill moving at rates of 2.0, 3.0 and 4.5 km/h for one minute measurement intervals. There was an about one minute pause between the subjects’ trials while the recording was saved and the next capture was prepared. Recording started after the subject’s gait pattern stabilized on the treadmill (usually after 5–10 seconds of stepping on the moving treadmill). The whole procedure was repeated with and without shoes.

#### 2.2.4. Accuracy of the virtual anatomical points

A marker set described in [22]–but without the heel markers—was used for virtual anatomical points (Fig 1). To measure the accuracy of the AR marker based system on the virtual anatomical points, an absolute comparison is required on their coordinates to the coordinates measured by the OptiTrack system. This requires a common reference system for the two measurements. Although the GoPro camera was placed with the optical axis parallel to the *x* axis of the OptiTrack system, this solution might not be completely accurate as discussed above. Furthermore, due to the closed structure of the camera, the exact location of the camera sensor—which is the center of the AR marker coordinate system—is difficult to align with the OptiTrack coordinate system. Another issue is the time synchronization of the data. As the two systems are not integrated, neither the shutter of the cameras nor the starting of the recording are synchronized. The previous will include a uniformly distributed error as much as the reciprocal of the sampling frequency. The later error—synchronization of the starting frames—can be eliminated similarly as the data is separated into gait cycles [23] by finding key frames in both datasets based on relative marker coordinates (peaks in the difference signal of a hip and an ankle virtual point coordinate). In order to move the virtual anatomical point coordinates measured by both systems in a common reference frame, the following data manipulation was performed:

- The starting time was synchronized by removing the beginning of both recordings before the starting frame of the fifth gait cycle of the right leg (S1A File).
- Based on the new common first frame, the gravity of both point clouds were moved to zero (S1B File).
- Based on the common first frame angular errors of the coordinate system axis, it was corrected in the AR marker measurements to match the coordinate system of the OptiTrack system. For this purpose, coordinate root mean square error optimization was performed to identify angular errors of the AR marker system.
- The gravity shifting (zero centering) transformation defined by the first frames was applied to the whole datasets. The angular correction based on the first frame of the AR dataset was applied for the whole AR dataset.

The first 500 frames of each virtual anatomical point coordinate in each measurement were concatenated for both systems grouped, by coordinate directions and the walking speed of the measurements. The accumulated OptiTrack and AR marker based data are finally compared using Bland-Altman plots.

#### 2.2.5. Calculation of gait parameters

The exported.trk file with the marker trajectories is used by the OpenSim program to run inverse kinematics on a musculoskeletal model (Gait2354). For each time step of recorded motion data, OpenSim computes a set of joint angles that put the model in a configuration that "best matches" the experimental kinematics. This "best match" is determined by solving a weighted least squares optimization problem with the goal of minimizing marker error. Marker error is defined as the distance between an experimental marker (virtual anatomical points in our terms) and the corresponding model marker placed on the OpenSim model anatomical points. The explanation of the joint angle calculation is summarized in the original paper of Delp et al. [24]. Contiguous motion is separated into gait cycles similarly to the method described in [23] at the peaks of coordinate *x* differences in the forward direction of the anterior superior iliac spine and the medial ankle. All compared parameters average values for a test case of the subjects’ gait cycles. The calculated spatial and angular parameters are described in Table 2. The range of motion (ROM) is defined for the angular gait variables. This is the difference of the maximum and minimum values of the joint trajectory. The processing of gait cycles, OpenSim joint data and spatiotemporal gait parameters are calculated by a custom Matlab script in Matlab version R2017b (MathWorks, Natick, Massachusetts, USA).

**Table 2:** Calculated gait parameters.

Parameter name / dimension	Definition
Stride length [m]	Distance by which each foot is in front of the other one at heel strike. Measured by medial ankle coordinates.
Step length [m]	Distance by which the foot moves forward in one gait cycle. Measured by medial ankle coordinates.
Walking base [m]	The side to side distance between the line of the two feet. Measured by medial ankle coordinates.
Cadence [steps/minute]	The total number of gait cycles taken within a minute. Calculated from the average cycle time of the individual gait cycles.
Hip flexion ROM [°]	Range of motion (difference of the maximum and minimum values of the joint angle trajectories) of the angular parameters averaged for the gait cycles of the trial as calculated by the OpenSim model described in [24].
Hip addiction ROM [°]	
Hip rotation ROM [°]	
Knee angle ROM [°]	
Pelvis tilt ROM [°]	
Pelvis list ROM [°]	
Pelvis rotation ROM [°]	
Pelvis tx ROM [m]	Range of translational motion (difference of the maximum and minimum coordinates) of the pelvis center coordinates averaged for the gait cycles of the trial as calculated by the OpenSim model described in [24].
Pelvis ty ROM [m]	
Pelvis tz ROM [m]	

The studied values are the mean values of the multiple gait cycles for each trials

ROM: range of motion, difference of the maximum and minimum values of the joint angle trajectories

#### 2.2.6. Statistical analysis

The calculated gait parameters were compared between the measurement systems. As each parameter was calculated for each trial and data recording was simultaneous on the two systems, the datasets could be paired. Root mean square errors (RMSE) for each averaged parameter were calculated between the datasets to characterize the accuracy of the AR marker system. Additionally, a Bland-Altman analysis [25] was conducted on these datasets to characterize correlation, limits of agreement on a 95% confidence interval, mean error and a reproducibility coefficient (RPC = 1.96SD) between the measurement systems. Additionally minimal detectable change (MDC) was calculated from the within-subject gait variability for both systems as:

$$MDC\;=\;SEM\times 1.962\times \sqrt{2}$$

where SEM is the standard error of measurement calculated as

$$SEM\;=\;\sqrt{\frac{\sum_{i\;=\;0}^{n}{SD}_{i}^{2}}{n}}$$

where *i* iterates over the measurements, *n* is the number of measurements, and *SD* is the standard deviation of the gait parameters for the individual gait cycles within the *i*-th measurement. While SEM is frequently calculated from the intraclass correlation coefficient, Baker [26] recommends the above simple method for calculating SEM to describe within-subject variability in gait analysis.

## 3. Results

### 3.1. Sample size

The measurements of two subjects had to be excluded from the study later on due to improper marker placement which was realized during the evaluation of the results. The elderly subject failed to perform the 4.5 km/h trials. The final number of trials therefore is *n* = 46 which includes trials from eight subjects with and without shoes at 2.0, 3.0 and 4.5 km/h walking speeds except the elderly subject where only 2.0 and 3.0 km/h trials were performed. This sample size produces 0.25SD standard error in the evaluation of the limit of agreement values in the Bland Altman analyses (1.71SD/√*n* according to Bland and Altman [25]).

### 3.2. Accuracy of virtual anatomical points

The summarized results of the Bland-Altman analysis for the virtual anatomical point position comparison are shown in Table 3. The results can be analyzed separately in each of the three coordinates (see directions on Fig 1) and walking speeds. The averaged slope of the regression lines was 1.05, 1.0 and 1.02 in directions *x*, *y*, and *z*, respectively, while the averaged *r*^*2*^ value was 0.98, 1.0, and 0.99, respectively. Due to the data manipulation to align the two reference frames, no significant bias can be observed on any coordinates. RPC values are the largest in direction x (mean: 33.92, SD: 3.42). The Bland-Altman plots of the analysis for each coordinate of the anatomical points grouped by directions and speed can be found in S2 File.

**Table 3:** Results of the Bland-Altman analysis on coordinates of virtual anatomical landmarks.

Anatomical landmark coordinates		r^2^	Slope	RPC (mm)	Mean error (mm)	95% confidence interval* (mm)
2 km/h	x	0.98	1.06	32.6	-0.05	(-33, 33)
	y	1	1	24.24	-1.6	(-26, 23)
	z	0.99	1.02	13.97	0.61	(-13, 15)
3 km/h	x	0.98	1.05	31.35	1	(-30, 32)
	y	1	1	26.42	-0.03	(-26, 26)
	z	1	1.02	11.97	1.5	(-11, 13)
4.5 km/h	x	0.98	1.05	37.8	1.6	(-36, 39)
	y	1	1	28.82	-0.2	(-29, 29)
	z	0.99	1.02	14.77	2.6	(-12, 17)
Mean (SD)	x	0.98 (0)	1.05 (0.01)	33.92 (3.42)	0.85 (0.84)	
	y	1 (0)	1 (0)	26.49 (2.29)	-0.61 (0.86)	
	z	0.99 (0.01)	1.02 (0)	13.57 (1.44)	1.57 (1.0)	

* 95% confidence interval equals the range of the bias ± 1.96 times the standard deviation of the differences. It is also referred to as the limit of agreement.

### 3.3. Deviation of gait parameters

**Fig 2:**
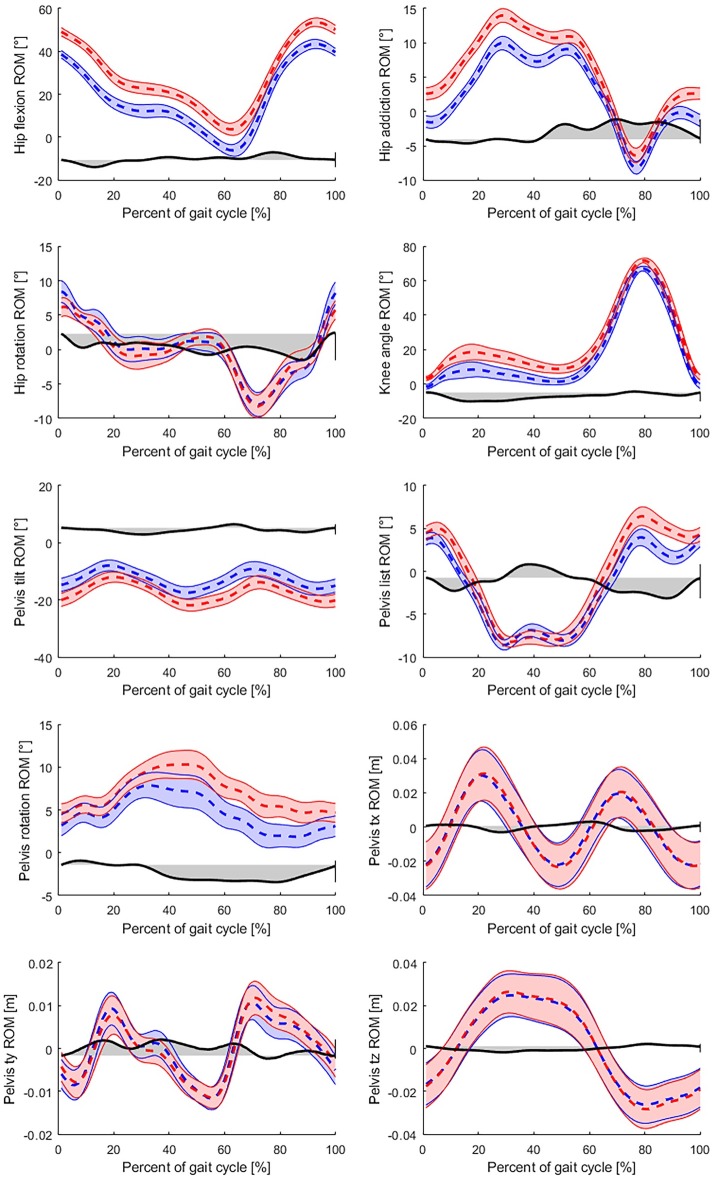
Comparison of joint angle trajectories. Joint trajectories measured by the AR marker based system (red) are drawn on top of the trajectories measured by the OptiTrack (blue). The dashed lines are the averaged joint trajectories during the trial, while the band around them is the ± intra-subject standard deviation at each percent of the gait cycle representing the gait variability. Differences of the two mean trajectories (black) are also illustrated in the figures. Due to camera position offset, the pelvis tx, ty and tz position parameters are zero centered for easier comparison. The range of motion gait parameters are defined by the difference of the maximum and minimum values of the averaged joint trajectories.

Detailed results of the Bland-Altman analysis, RMSE and MDC values are presented in Table 4 for each calculated gait parameter. The corresponding Bland-Altman- and correlation diagrams can be found in S3 File. Overall, distance type parameters showed RMSE as smaller than or equal to 23 mm with a mean error (bias) smaller than or equal to 7 mm and CV smaller than 4.6%. The mean error of the angle type ROM parameters indicated significant deviations between the two measurement systems with a mean error range between 1.27° and 3.91° and an RMSE range between 2.55°and 6.72°. The detection of the pelvis position range of motion also showed small mean errors (≤ 1 mm) with RMSE between 5 and 8 mm, but larger CV (16.5–19.4%). The only time based parameter of cadence (step frequency) yielded a mean error of 0.18 steps/minute with RMSE of 1.116 steps/minute and 1.19% CV. Most RMS errors are in the range of MDC of the OptiTrack system. In case of hip adduction and rotation ROM and pelvis list and rotation angles the RMS error is larger than the MDC values. For illustrating the differences between the measurement systems, joint angle trajectories of a subject in the 2 km/h trial are shown in Fig 2, where it is visible that mean differences in certain parameters are in the range of gait variability (hip rotation, pelvis list and positions), while other parameters have significant offset errors (pelvis rotation and tilt, hip flexion and addiction).

**Table 4:** RMS error and Bland-Altman analysis of gait parameters.

Parameter	RMSE	Bland-Altman analysis						MDC (Opti-Track)	MDC (AR)
		r^2^	Slope	RPC	CV (%)	Mean error	95% confidence interval of error		
Stride length [m]	0.013	0.996	0.988	0.026	1.201	-0.002	(-0.028; 0.024)	0.059	0.052
Step length [m]	0.023	0.956	0.996	0.044	4.105	-0.007	(-0.050; 0.037)	0.060	0.066
Walking base [m]	0.023	0.915	0.947	0.016	4.594	-0.003*	(-0.019; 0.013)	0.049	0.040
Hip flexion ROM [°]	4.666	0.848	0.898	8.848	12.081	1.274*	(-7.574; 10.122)	6.725	6.629
Hip addiction ROM [°]	3.489	0.647	0.764	5.129	17.306	2.324*	(-2.805; 7.452)	3.398	3.442
Hip rotation ROM [°]	6.728	0.459	0.687	10.792	34.586	3.910*	(-6.882; 14.701)	3.699	3.509
Knee angle ROM [°]	3.607	0.945	1.040	6.555	5.809	1.396*	(-5.159; 7.952)	4.283	4.487
Pelvis tilt ROM [°]	2.554	0.558	1.128	4.388	34.497	1.272*	(-3.116; 5.660)	3.779	4.179
Pelvis list ROM [°]	3.750	0.297	0.518	6.761	33.792	1.556*	(-5.205; 8.316)	2.432	4.067
Pelvis rotation ROM [°]	3.678	0.641	0.887	6.004	31.249	-2.086*	(-8.090; 3.918)	3.487	4.922
Pelvis tx ROM [m]	0.008	0.573	0.701	0.016	19.483	<0.001	(-0.015; 0.016)	0.021	0.023
Pelvis ty ROM [m]	0.005	0.888	1.243	0.010	16.159	0.001	(-0.008; 0.011)	0.008	0.009
Pelvis tz ROM [m]	0.008	0.713	0.813	0.016	16.585	<0.001	(-0.016; 0.016)	0.019	0.020
Cadence [steps/minute]	1.116	0.995	1.010	2.180	1.191	0.187	(-1.993; 2.368)	-	-

*Significant mean error (p<0.05),

ROM: range of motion, RMSE: root mean square error, r^2^: squared Pearson r-value of the correlation plot, Slope: the slope RPC: reproducibility coefficient (1.96*SD), CV: coefficient of variation (SD of mean values in %)

## 4. Discussion

The paper described the concept and possible technical solutions of a gait analysis system using a single action camera and AR markers, and its aim was to validate an exemplified implementation of the concept with simultaneous measurements of a conventional motion capture system. A validation of the system has been performed by an 18 camera OptiTrack motion capture system on healthy gait at different walking speeds (2.0, 3.0 and 4.5 km/h) on a treadmill and by comparing 3D anatomical point coordinates (Table 3) and several gait parameters (Tables 2 and 4) using both systems. Generally, significant mean errors of angular ROM gait parameters (marked with * in Table 4) can be observed in the AR marker system; however, the errors of the distance type parameters are relatively smaller, except for the walking base.

Studying the absolute errors of virtual anatomical points (Table 3), it is obvious that absolute coordinate errors depend on the direction (Fig 1), as the reproducibility coefficients (RPC)—especially in the direction of motion (*x)* and also in the upward (*y)* direction—are larger than errors in the medial-lateral (*z)* direction. These errors are also larger at higher walking speed where a larger range of motion can be observed (Table 3). These errors can be traced back to the calculation of marker orientations. The coordinate system convention for understanding this explanation is shown in Fig 1 as axis *x* points in the forward direction of the movement, *y* points upward, and *z* points to the right. The orientation of the markers with respect to the camera is calculated through homography transformation from the pixel coordinates of the marker corners [19]. In the applied orientations of the markers, the sideway accuracy of virtual anatomical points is mostly influenced by the accuracy of marker rotation around axis *x* and the marker position in direction *z*, which can be highly accurately calculated from the high resolution image of a properly calibrated camera. On the other hand, marker rotations around axes *y* and *z* and the *x* coordinate calculation of the marker are more influenced by camera perspective due to homography transformations.

On the other hand, the angular errors of the AR marker detection affect more the observed joint angles (e.g. knee angle and pelvis flexion) and the virtual ankle *x* coordinates that are used to calculate step length and stride length (Table 3). This suggests that the location of the camera may affect the recording quality, and these joint angles might be more accurate when the gait analysis is done in the sagittal plane (the camera and AR markers are on the side of the subject). In this setup only one leg can be analyzed. Preliminary results with the same measurement setup showed that unfortunately the results are not significantly better this way [27], as all the markers cannot face the camera continuously and even in stationary position the markers do not line in a plane which is perpendicular to the optical axis and the orientation estimation is also introduce errors in this setup. The 3D motion capture is performed regardless of the camera position and joint angles can be captured in each plane of the motion.

The between-system differences for some parameters are in the range of differences between stereophotogrammetric motion capture systems such as the one used as reference in the present study (the OptiTrack camera system). Thewlis et al. found 2.7° mean differences in knee range of motion between two commercial motion capture camera systems in a simultaneously measured gait trial [28]. This is comparable to our even smaller deviation in knee range of motion (1.39°). The proposed system can accurately measure human gait with maximum 3.91° mean error (hip rotation ROM) in the studied angular parameters and maximum 7 mm mean error in spatial parameters. On the other hand, higher RMSE values show us that the deviations are nondeterministic, therefore longer or multiple averaged measurements are required for calculating averaged gait parameters for walking trials.

The calculated MDC values of both system are of similar values to those published in the literature (e.g. in Fernandes et al. [29] stride length: 0.09 m, step length: 0.05 m, step width: 0.02 m, and peak parameters for hip flexion 7.9°, hip adduction 3.9°, knee angle 5.5–7°, or in Bates et al. [30] ROM parameters for hip flexion: 2.51°, hip adduction 1.48°, hip rotation: 4.35°, knee angle: 5.34°, pelvis tilt: 1.34°, pelvis rotation: 1.88°). Most RMS errors are in the range of MDC of the OptiTrack system, thus this difference is not statistically significant when deviations from a healthy reference group is sought. In case of hip adduction and rotation, pelvis list and rotation angles the RMS error is larger than the MDC values and even in the pelvis angles MDC is slightly (0.4–1.6 degree) larger than in the OptiTrack system. In this case repeated measurements between the two systems on the same patient would show deviations. On the other hand other MDC values by the two system are very similar (Table 4).

Compared to gait analysis reports from the literature [1,31–33], within-subject and inter-subject differences could be shown with these errors by the proposed system in common gait analysis applications such as the following examples. Kim and Eng [31] have studied inter-subject angular differences in the paretic and non-paretic legs of stroke survivors. For the knee flexion ROM they found 16.1° mean difference between legs [31], while the RMS error of the knee flexion ROM is 3.6° between the AR marker system and OptiTrack, thus this deviation could have been shown by the proposed system. Bejek et al. have studied the effect of walking speed on gait parameters in patients with osteoarthritis and healthy controls [1]. In their study, step size differs by more than 200 mm in each group between different walking speeds (1, 2, 3 and 4 km/h), while the RMS error of this parameter of our system is only 23 mm. The asymmetry of step length in the osteoarthritis group is between 38.1 and 217 mm for the different walking speeds. Similarly, the asymmetry of the knee angle ranges between 6.4° and 15.9° at 1 and 4 km/h walking speed [1], while the RMS of knee angle is 3.6 with our system. Derrick [32] collected knee angle measurements from the literature at foot contact with different experimental procedures: knee contact angle changes between 10% understride and 10% overstride by 2°, due to fatigue by 4.4°, between smooth and irregular walking surface by 1.5°, and between short and long grass on the walking surface by 4.2°. Duffel and Jordan found an insignificant 2.5° difference in the largest knee angle between 18–30 and 60+ year old healthy subjects [33]. The above examples demonstrate multiple use cases of gait analysis where the present gait parameters are used with smaller (mostly statistically not significant) or larger differences between cases compared to the measurement errors of our system. The very small differences cannot be reliably measured with the present exemplified system, but significant deviations could be observed with even the present rudimentary implementation of an AR marker based motion capture. At this point it is important to consider that Thewlis et al found 2.7° mean difference of the knee ROM between commercially available multi camera motion capture systems [28]. The usability of the present (and any) system depends on the expected effect size of research.

Although the exemplified implementation of the proposed approach does not yet fulfil all requirements expected from the high-end user friendly multi-camera motion capture system, the proposed approach can be utilized in the development of a consumer grade low cost motion capture system by refining some of the technological cornerstones, e.g. more precise camera calibration, more precise AR marker tracking and real-time behavior. The most important upgrade could be the improvement of marker orientation precision. One solution could be the usage of a different augmented reality marker using a microlense array which promises higher orientation accuracy [34].

## 5. Conclusion

The study introduced a new, mobile and affordable gait analysis approach using augmented reality markers fixed on body segments recorded by an action camera. The solution was introduced and validated using an OptiTrack motion capture system with multiple walking speeds and subjects. The proposed method shows some differences in the raw coordinates of virtually tracked anatomical landmarks (RPC 33.92 mm in direction *x*, 26.49 in direction *y* and 13.57 mm in direction *z*) and gait parameters compared to the reference system (Table 4); however, these differences are comparable to previously reported differences between commercial motion capture systems. Accuracy might be improved by more advanced AR marker tracking.

## Supporting information

S1 File Data manipulation figures. (PDF)

S2 File Bland-Altman plots for virtual anatomical points. (PDF)

S3 File Bland-Altman plots of gait parameters. (PDF)

S4 File Gait parameters comparison data. (XLSX)

S5 File C# source code snippets for anatomical calibration and anatomical point tracking using homogeneous coordinate transformation. (PDF)

S1 Fig Anatomical point calibration animation. (GIF)

S2 Fig Gait analysis with AR markers animation. (GIF)
