# Supplementary material for: Affordable gait analysis using augmented reality markers
Source: PLoS One. 2019 Feb 14;14(2):e0212319. doi: 10.1371/journal.pone.0212319 (PMC6375625; doi:10.1371/journal.pone.0212319)

A

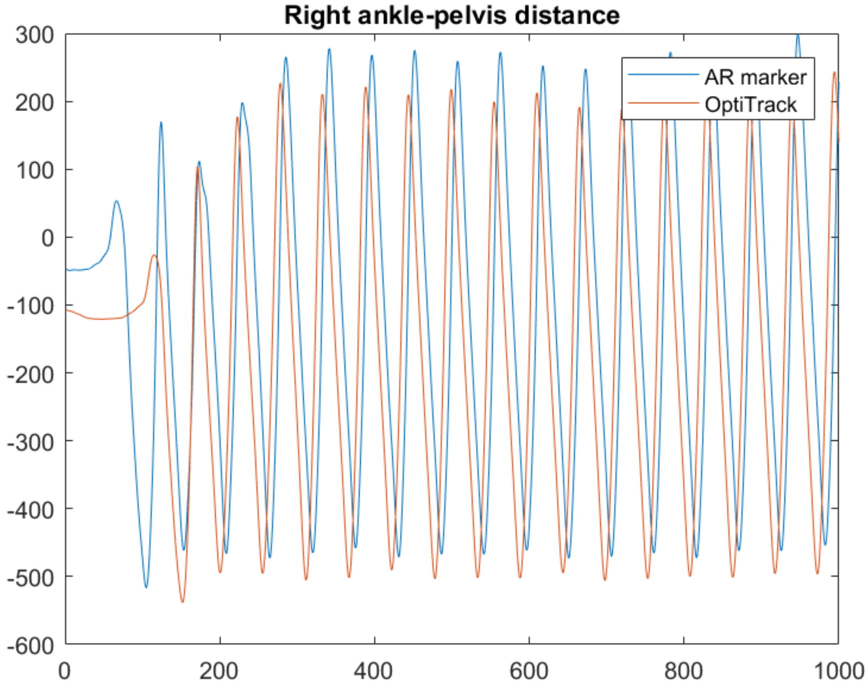

The starting time was synchronized by removing the beginning of both recordings before the starting frame of the fifth gait cycle of the right leg.

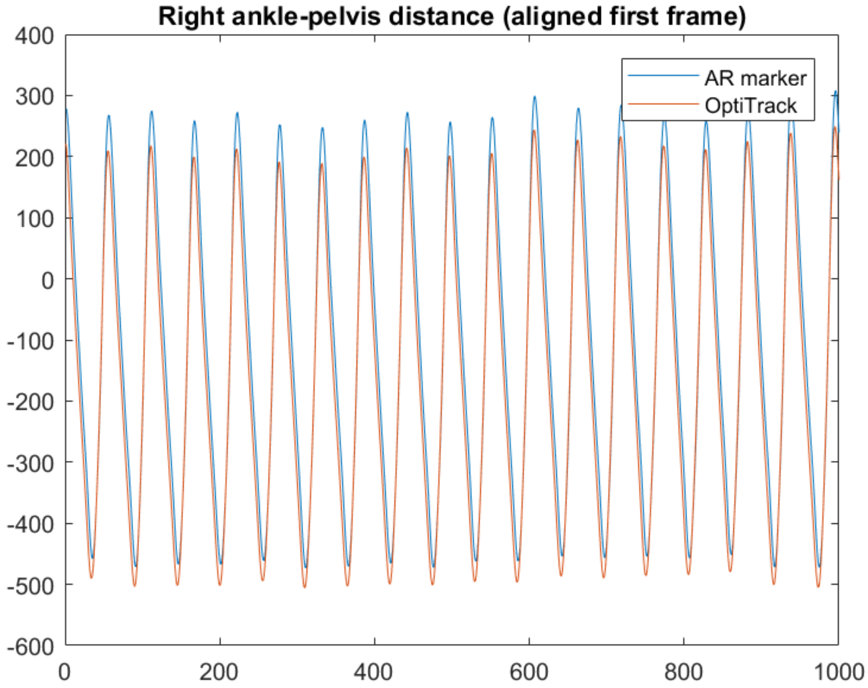

B

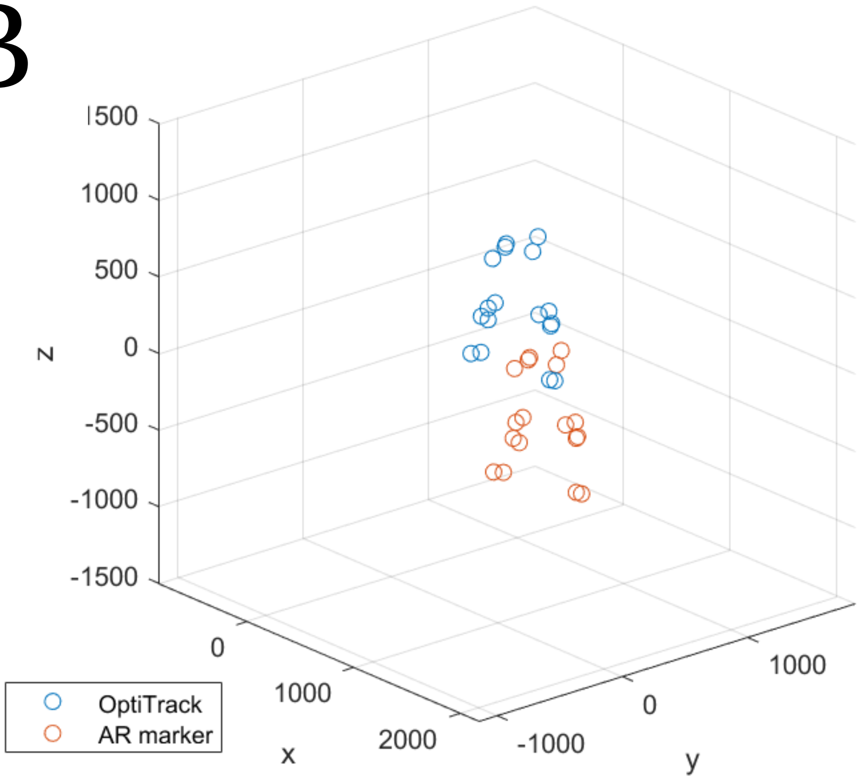

Based on the new common first frame, the gravity of both point clouds were moved to zero.

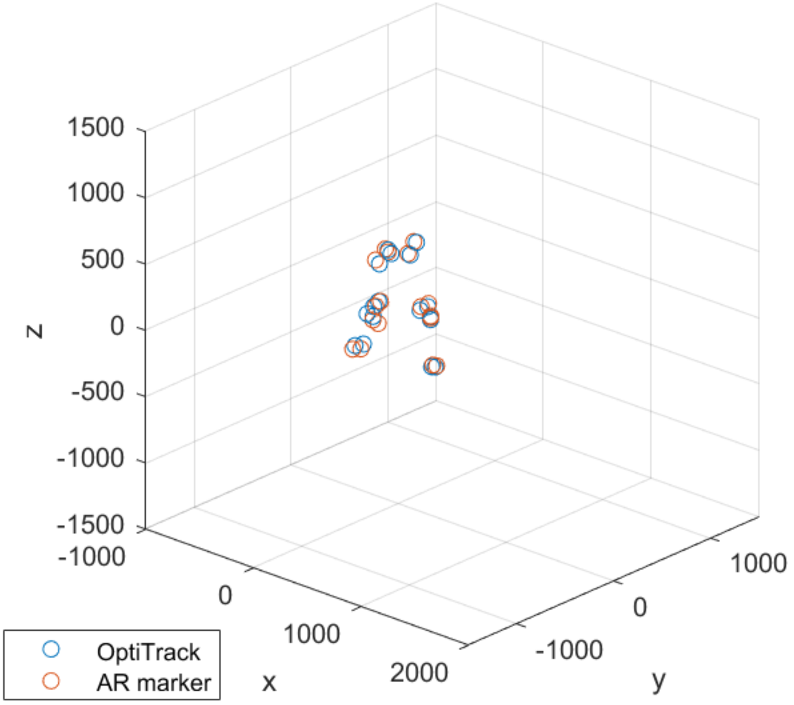

Supplement: S1 File — (PDF) [file pone.0212319.s001.pdf]
