# Supplementary material for: Affordable gait analysis using augmented reality markers
Source: PLoS One. 2019 Feb 14;14(2):e0212319. doi: 10.1371/journal.pone.0212319 (PMC6375625; doi:10.1371/journal.pone.0212319)
Supplement: S2 File — (PDF) [file pone.0212319.s002.pdf]

X coordinates (2km/h)

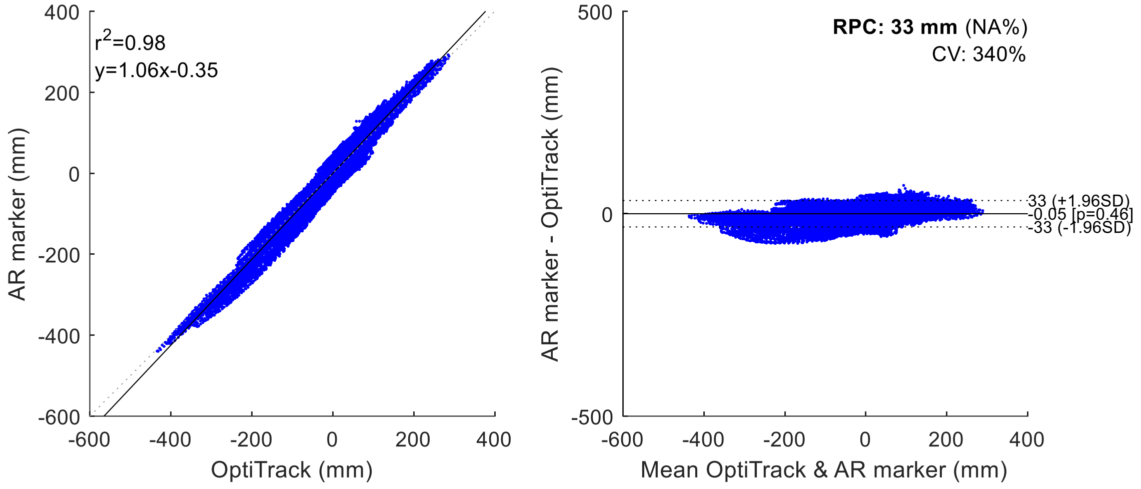

Y coordinates (2km/h)

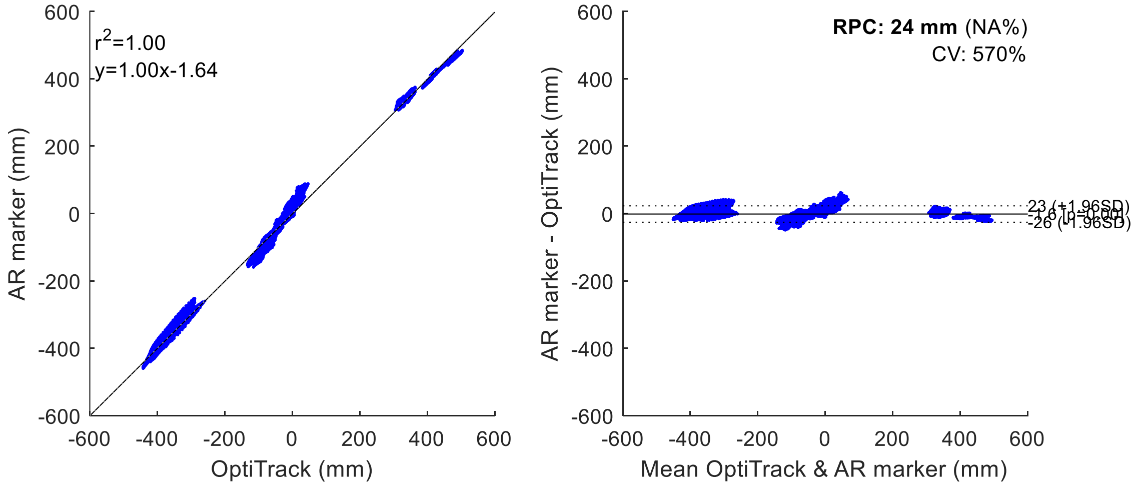

Z coordinates (2km/h)

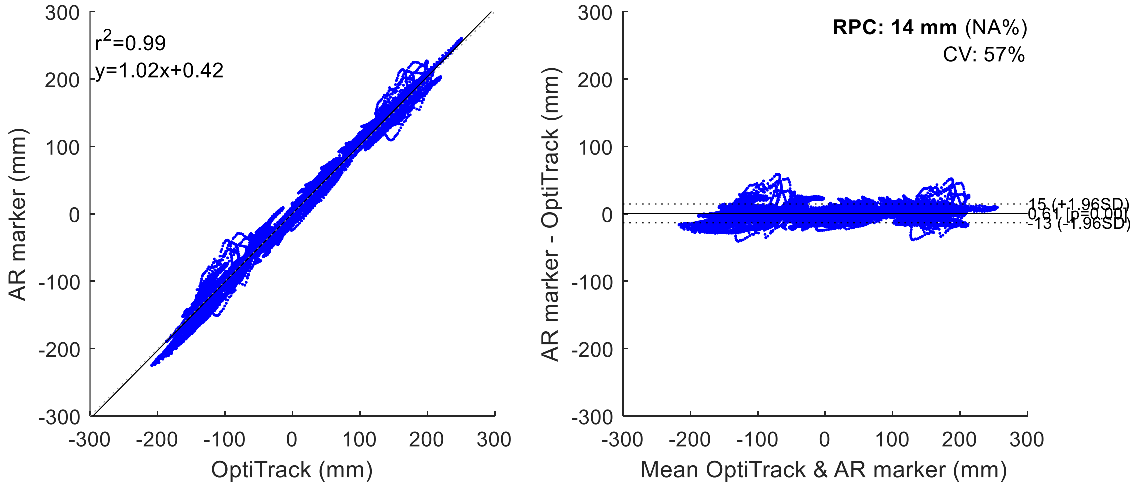

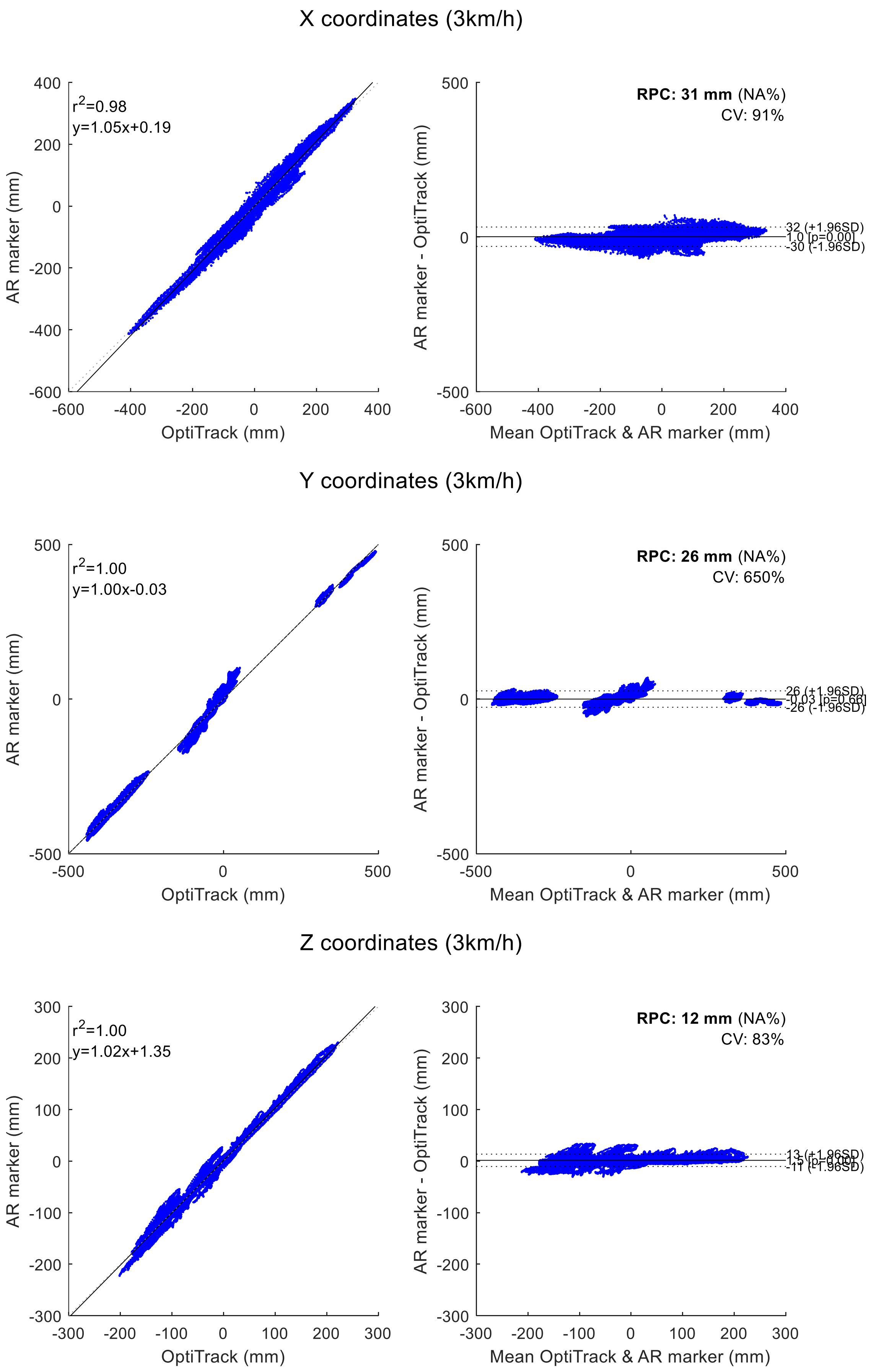
X coordinates (3km/h)
r2=0.98
y=1.05x+0.19
AR marker (mm)
OptiTrack (mm)
RPC: 31 mm (NA%)
CV: 91%
32 (+1.96SD)
1.0 [p=0.00]
-30 (-1.96SD)
AR marker - OptiTrack (mm)
Mean OptiTrack & AR marker (mm)
Y coordinates (3km/h)
r2=1.00
y=1.00x-0.03
AR marker (mm)
OptiTrack (mm)
RPC: 26 mm (NA%)
CV: 650%
26 (+1.96SD)
-0.03 [p=0.66]
-26 (-1.96SD)
AR marker - OptiTrack (mm)
Mean OptiTrack & AR marker (mm)
Z coordinates (3km/h)
r2=1.00
y=1.02x+1.35
AR marker (mm)
OptiTrack (mm)
RPC: 12 mm (NA%)
CV: 83%
13 (+1.96SD)
-11 (-1.96SD)
AR marker - OptiTrack (mm)
Mean OptiTrack & AR marker (mm)

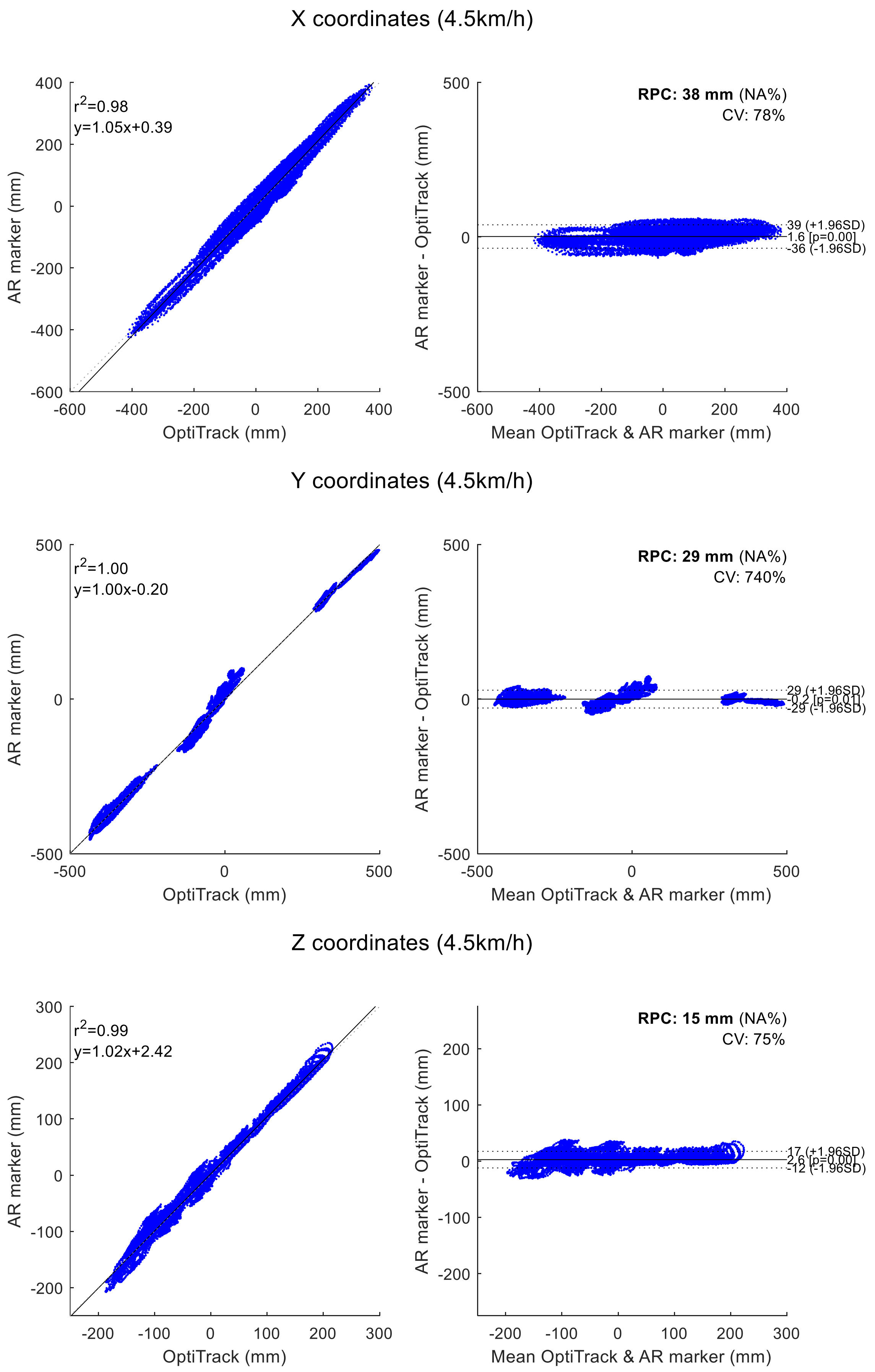

X coordinates (4.5km/h)
r^2=0.98
y=1.05x+0.39
AR marker (mm)
OptiTrack (mm)
RPC: 38 mm (NA%)
CV: 78%
39 (+1.96SD)
1.6 [p=0.00]
-36 (-1.96SD)
AR marker - OptiTrack (mm)
Mean OptiTrack & AR marker (mm)
Y coordinates (4.5km/h)
r^2=1.00
y=1.00x-0.20
AR marker (mm)
OptiTrack (mm)
RPC: 29 mm (NA%)
CV: 740%
29 (+1.96SD)
-0.2 [p=0.01]
-29 (-1.96SD)
AR marker - OptiTrack (mm)
Mean OptiTrack & AR marker (mm)
Z coordinates (4.5km/h)
r^2=0.99
y=1.02x+2.42
AR marker (mm)
OptiTrack (mm)
RPC: 15 mm (NA%)
CV: 75%
17 (+1.96SD)
2.6 [p=0.00]
-12 (-1.96SD)
AR marker - OptiTrack (mm)
Mean OptiTrack & AR marker (mm)
