# Supplementary material for: Affordable gait analysis using augmented reality markers
Source: PLoS One. 2019 Feb 14;14(2):e0212319. doi: 10.1371/journal.pone.0212319 (PMC6375625; doi:10.1371/journal.pone.0212319)
Supplement: S3 File — (PDF) [file pone.0212319.s003.pdf]

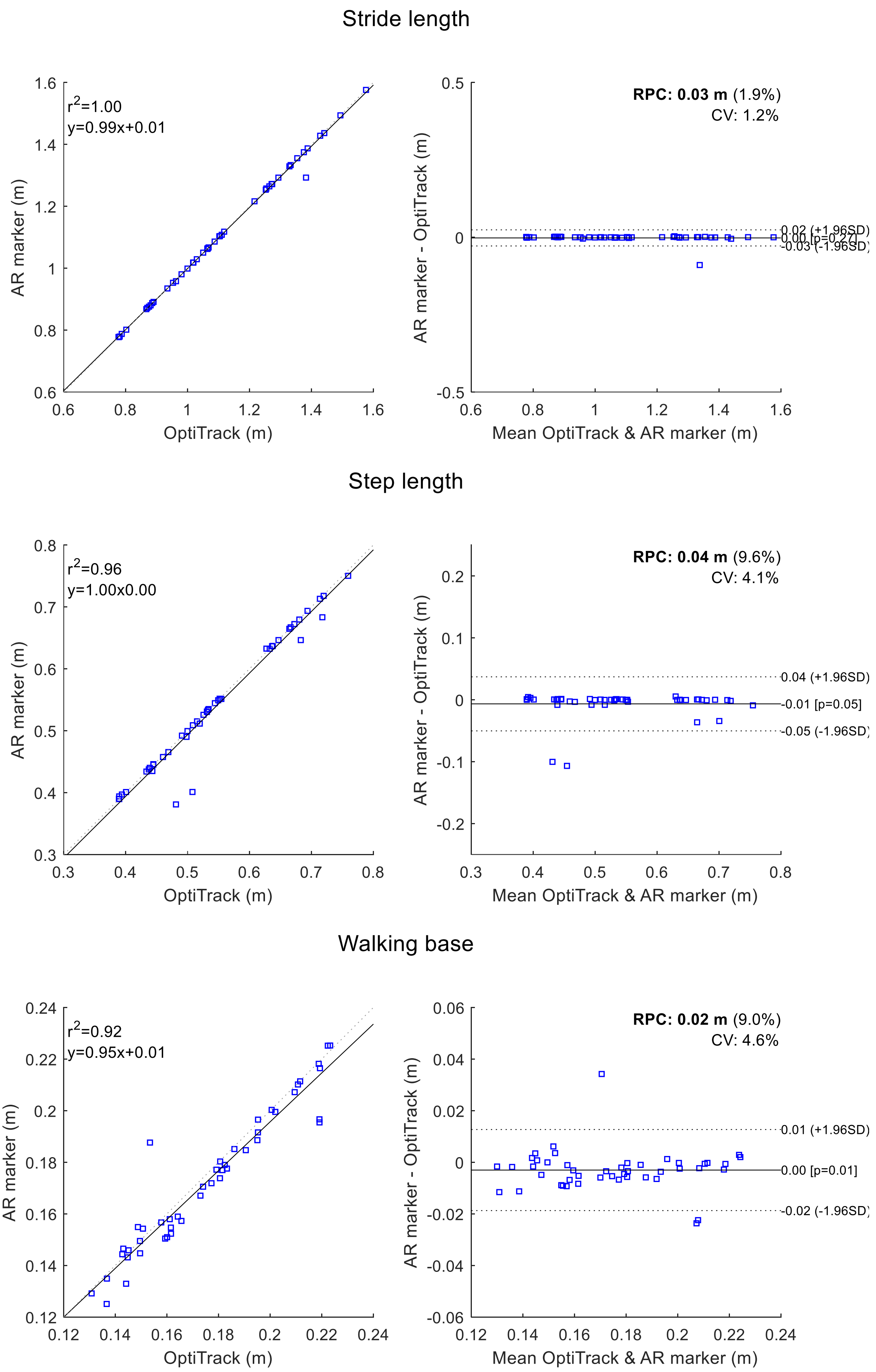

Stride length
r²=1.00
y=0.99x+0.01
AR marker (m)
OptiTrack (m)
RPC: 0.03 m (1.9%)
CV: 1.2%
0.02 (+1.96SD)
0.00 [p=0.27]
-0.03 (-1.96SD)
AR marker - OptiTrack (m)
Mean OptiTrack & AR marker (m)
Step length
r²=0.96
y=1.00x0.00
AR marker (m)
OptiTrack (m)
RPC: 0.04 m (9.6%)
CV: 4.1%
0.04 (+1.96SD)
-0.01 [p=0.05]
-0.05 (-1.96SD)
AR marker - OptiTrack (m)
Mean OptiTrack & AR marker (m)
Walking base
r²=0.92
y=0.95x+0.01
AR marker (m)
OptiTrack (m)
RPC: 0.02 m (9.0%)
CV: 4.6%
0.01 (+1.96SD)
0.00 [p=0.01]
-0.02 (-1.96SD)
AR marker - OptiTrack (m)
Mean OptiTrack & AR marker (m)

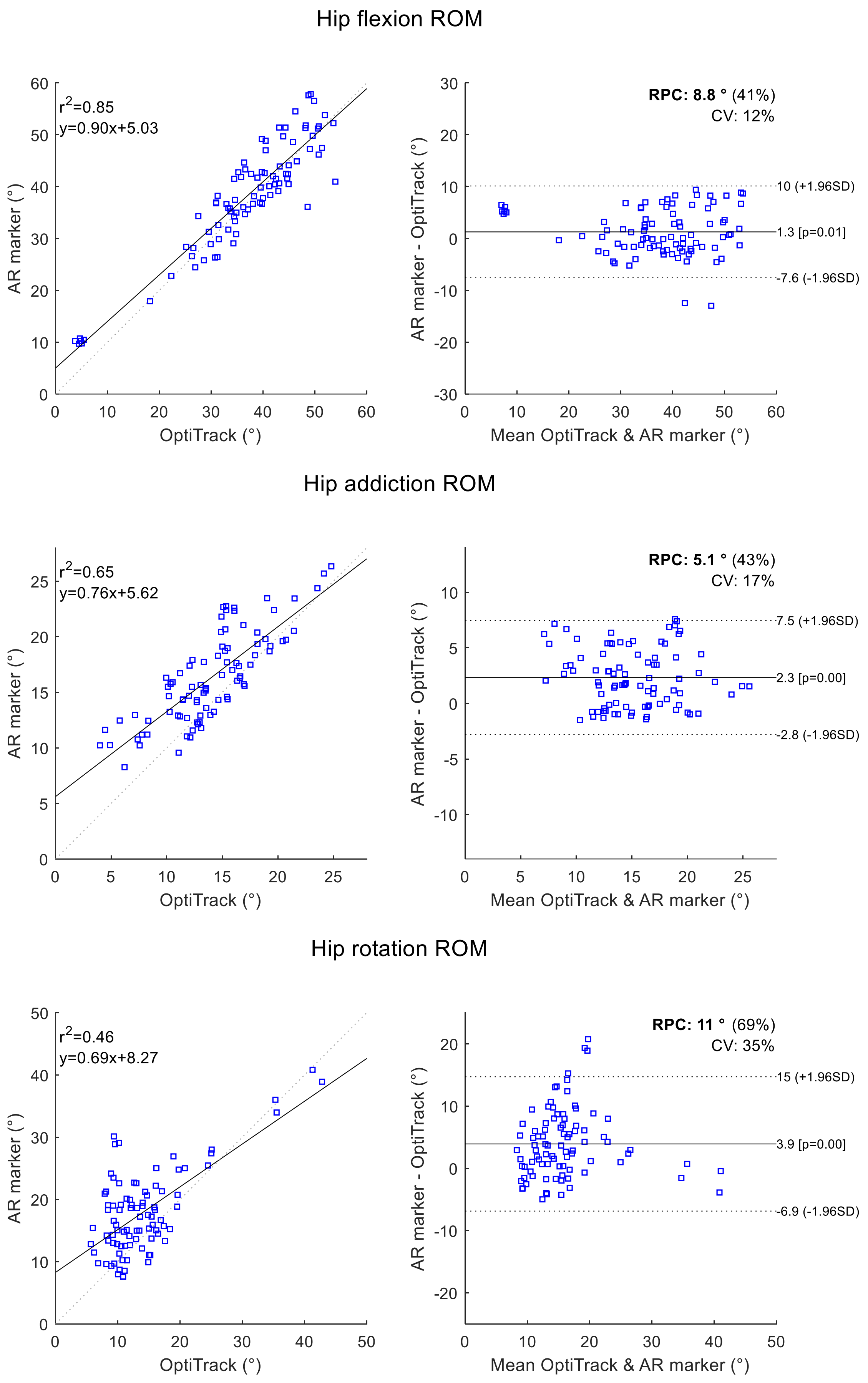

Hip flexion ROM
r²=0.85
y=0.90x+5.03
AR marker (°)
OptiTrack (°)
RPC: 8.8 ° (41%)
CV: 12%
10 (+1.96SD)
1.3 [p=0.01]
-7.6 (-1.96SD)
AR marker - OptiTrack (°)
Mean OptiTrack & AR marker (°)
Hip addiction ROM
r²=0.65
y=0.76x+5.62
AR marker (°)
OptiTrack (°)
RPC: 5.1 ° (43%)
CV: 17%
7.5 (+1.96SD)
2.3 [p=0.00]
-2.8 (-1.96SD)
AR marker - OptiTrack (°)
Mean OptiTrack & AR marker (°)
Hip rotation ROM
r²=0.46
y=0.69x+8.27
AR marker (°)
OptiTrack (°)
RPC: 11 ° (69%)
CV: 35%
15 (+1.96SD)
3.9 [p=0.00]
-6.9 (-1.96SD)
AR marker - OptiTrack (°)
Mean OptiTrack & AR marker (°)

Knee angle ROM

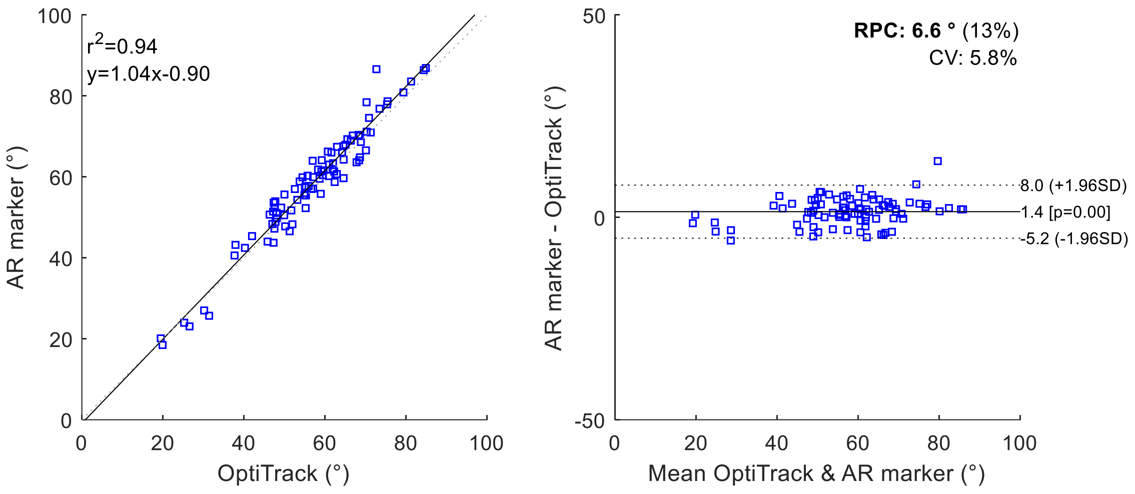

Pelvis tilt ROM

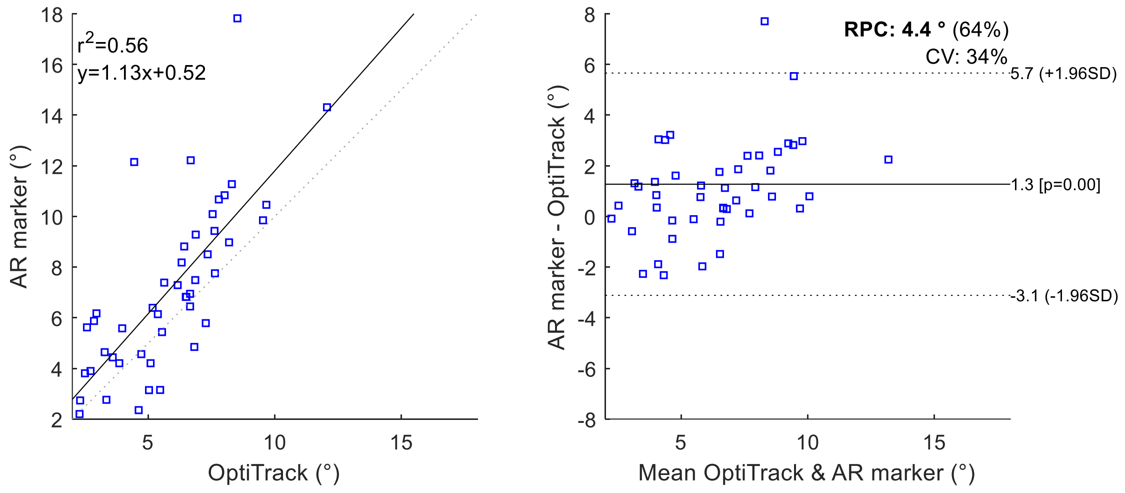

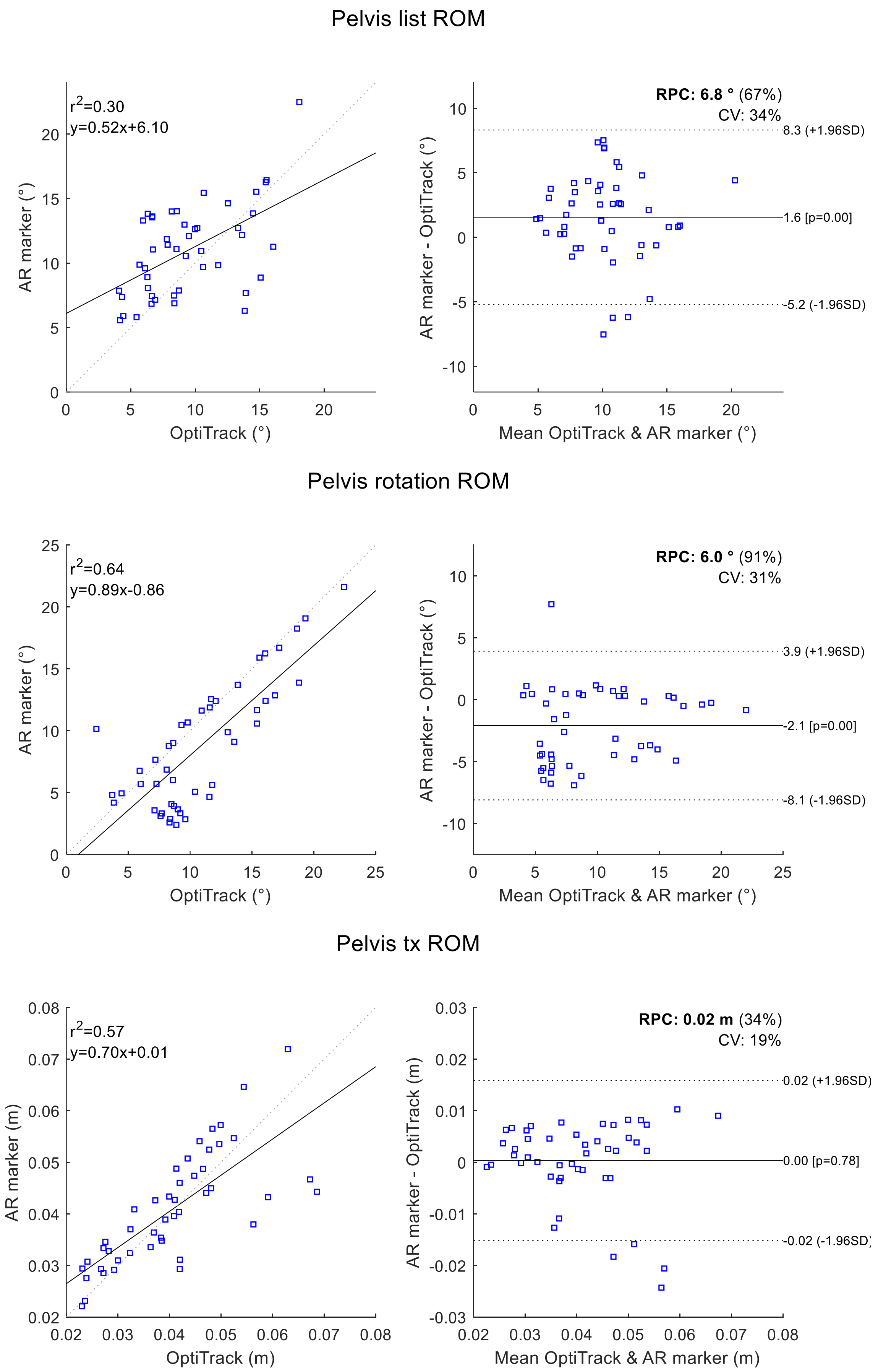

Pelvis list ROM
r2=0.30
y=0.52x+6.10
AR marker (°)
OptiTrack (°)
RPC: 6.8 ° (67%)
CV: 34%
8.3 (+1.96SD)
1.6 [p=0.00]
-5.2 (-1.96SD)
AR marker - OptiTrack (°)
Mean OptiTrack & AR marker (°)
Pelvis rotation ROM
r2=0.64
y=0.89x-0.86
AR marker (°)
OptiTrack (°)
RPC: 6.0 ° (91%)
CV: 31%
3.9 (+1.96SD)
-2.1 [p=0.00]
-8.1 (-1.96SD)
AR marker - OptiTrack (°)
Mean OptiTrack & AR marker (°)
Pelvis tx ROM
r2=0.57
y=0.70x+0.01
AR marker (m)
OptiTrack (m)
RPC: 0.02 m (34%)
CV: 19%
0.02 (+1.96SD)
0.00 [p=0.78]
-0.02 (-1.96SD)
AR marker - OptiTrack (m)
Mean OptiTrack & AR marker (m)

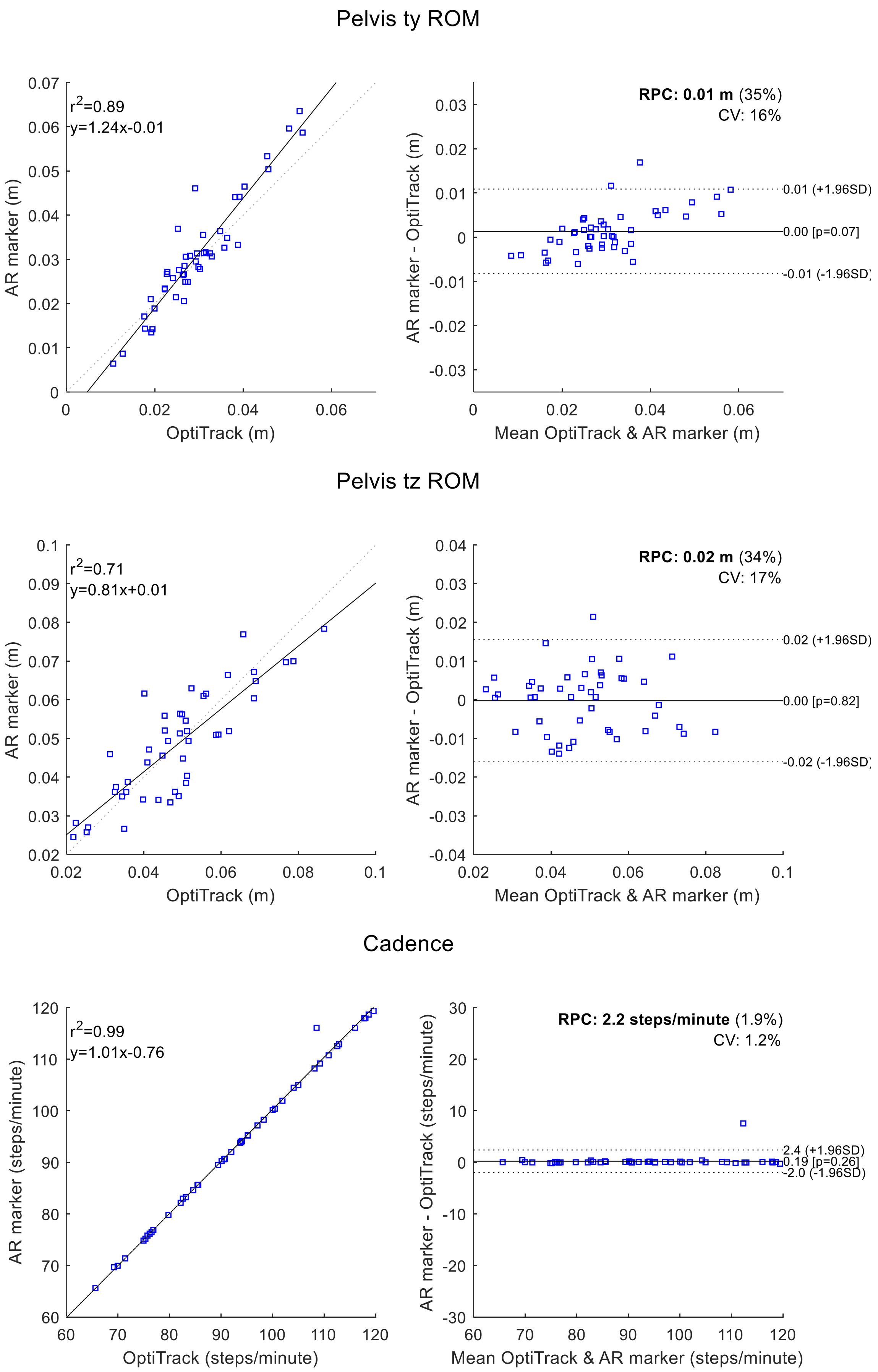
Pelvis ty ROM
r2=0.89
y=1.24x-0.01
AR marker (m)
OptiTrack (m)
RPC: 0.01 m (35%)
CV: 16%
0.01 (+1.96SD)
0.00 [p=0.07]
-0.01 (-1.96SD)
AR marker - OptiTrack (m)
Mean OptiTrack & AR marker (m)
Pelvis tz ROM
r2=0.71
y=0.81x+0.01
AR marker (m)
OptiTrack (m)
RPC: 0.02 m (34%)
CV: 17%
0.02 (+1.96SD)
0.00 [p=0.82]
-0.02 (-1.96SD)
AR marker - OptiTrack (m)
Mean OptiTrack & AR marker (m)
Cadence
r2=0.99
y=1.01x-0.76
AR marker (steps/minute)
OptiTrack (steps/minute)
RPC: 2.2 steps/minute (1.9%)
CV: 1.2%
2.4 (+1.96SD)
0.19 [p=0.26]
-2.0 (-1.96SD)
AR marker - OptiTrack (steps/minute)
Mean OptiTrack & AR marker (steps/minute)
