# Supplementary material for: Affordable gait analysis using augmented reality markers
Source: PLoS One. 2019 Feb 14;14(2):e0212319. doi: 10.1371/journal.pone.0212319 (PMC6375625; doi:10.1371/journal.pone.0212319)
Supplement: S5 File — (PDF) [file pone.0212319.s005.pdf]

C# implementtion of anatomical point calculation and calibration

This supplementary material explains the process of anatomical calibration and virtual anatomical point tracking using augmented reality markers. First the relevant data classes are introduced, then the implementation of necessary mathematical formulas for homogeneous coordinate transformation (matrix rotations). Then the process of anatomical calibration is explained where the local coordinates of the virtual anatomical points are set with respect to the actually tracked body segment coordinates and orientations through the augmented reality marker tracking.

The **Segment** class represents a body segment defined by an AR marker position and orientation provided by any augmented reality framework, e.g. AprilTag AR marker tracking (downloadable from: https://april.eecs.umich.edu/software/apriltag)

```
public class Segment
    {
        public String name;

        public Vector<double> position;
        public Vector<double> orientation;  //ZXZ rotational euler conventions

        public bool isVisible;

        public Segment(String name)
        {
            this.name = name;

            position = Vector<double>.Build.Dense(3);
            orientation = Vector<double>.Build.Dense(3);

            isVisible = false;
        }
    }
```

The **Marker** class represents a virtual anatomical point that belongs to a certain body segment through its *segmentID* variable which refer to the index of the corresponding segment in an array of body segments that defines our biomechanical model.
The local coordinates refer to the anatomical point coordinates respect to the AR marker centered moving reference frame attached to the corresponding body segment where the anatomical point is defined and moves rigidly together with the segment.
The global coordinates of the anatomical points refer to their positions defined in the external reference frame (e.g. camera optical center point) in which the movement of the segments (AR markers) are defined and tracked. This global position is calculated through homogeneous coordinate transformation.

```
public class Marker
    {
        public String name;
        public int segmentID;

        public Vector<double> globalCoordinates;
        public Vector<double> localCoordinates;

        public bool isVisible;

        public Marker(String name, int segmentID)
        {
            this.name = name;
            this.segmentID = segmentID;

            globalCoordinates = Vector<double>.Build.Dense(3);
            localCoordinates = Vector<double>.Build.Dense(3);

            isVisible = false;
        }

        public Marker(Marker markerToCopy)
        {
            this.name = markerToCopy.name;
            this.segmentID = markerToCopy.segmentID;

            globalCoordinates =
Vector<double>.Build.DenseOfVector(markerToCopy.globalCoordinates);
            localCoordinates =
Vector<double>.Build.DenseOfVector(markerToCopy.globalCoordinates);

            isVisible = markerToCopy.isVisible;
        }

        public bool IsCalibrated()
        {
            if (localCoordinates[0] == 0 && localCoordinates[1] == 0 &&
localCoordinates[2] == 0)
                return false;
            else
                return true;
        }
    }
```

RotMatrices is a helper class that performs matrix operations with rotation matrices for calculating homogeneous coordinate transformation. In this class the ZXZ rotational convention is applied with respect to the order of the rotation axes.

```
public static class RotMatrices
{
    public static Matrix<double> X(double fi)
    {
        Matrix<double> R = DenseMatrix.OfArray(new double[,] {
        {1.0,0.0,0.0},
        {0.0,Math.Cos(fi),-Math.Sin(fi)},
        {0.0,Math.Sin(fi),Math.Cos(fi)}});

        return R;
    }

    public static Matrix<double> Y(double fi)
    {
        Matrix<double> R = DenseMatrix.OfArray(new double[,] {
        {Math.Cos(fi),0.0,Math.Sin(fi)},
        {0.0,1.0,0.0},
        {-Math.Sin(fi),0.0,Math.Cos(fi)}});

        return R;
    }

    public static Matrix<double> Z(double fi)
    {
        Matrix<double> R = DenseMatrix.OfArray(new double[,] {
        {Math.Cos(fi),-Math.Sin(fi),0.0},
        {Math.Sin(fi),Math.Cos(fi),0.0},
        {0.0,0.0,1.0}});

        return R;
    }

    public static Matrix<double> ZXZ(Vector<double> orientation)
    {
        Matrix<double> R = Z(orientation[0]) * X(orientation[1]) * Z(orientation[2]);
        return R;
    }

    public static Matrix<double> invZXZ(Vector<double> orientation)
    {
        Matrix<double> R = Z(-orientation[2]) * X(-orientation[1]) * Z(-
orientation[0]);
        return R;
    }
}
```

During the moment of **calibration of anatomical points** the instantaneous global coordinates of the anatomical point are given by the calibration point of the calibration wand which in this practice acts as a special segment. (In the recording this must be performed when - after palpation - the calibration point is aligned to the calibrated anatomical point of the subject.) The local coordinates of the calibration wand is known as by design, thus the calibration point can always be calculated.
From the instantaneous global coordinates of the anatomical point (given by the calibration point) and the actually tracked coordinates and orientation of the segment the local coordinates of the anatomical point relative to the tracked segment is calculated in the **UpdateLocalCoordinates** function.

```
public bool CalibrateAnatPoint(int markerID)
{
    Marker anatPoint = markerDictionary[markerID];

    if (bodySegmentDictionary.ContainsKey(anatPoint.segmentID))
    {
        Segment parentSegment = bodySegmentDictionary[anatPoint.segmentID];

        if (parentSegment.isVisible)
        {
            Marker calibrationWand = markerDictionary[GaitModel.POINTER_MARKER_ID];

            if (calibrationWand.isVisible)
            {
                UpdateLocalCoordinates(anatPoint, parentSegment, calibrationWand);
                return true;
            }
            else
            {
                throw new System.Exception("The calibration wand is not visible. The
anatomical point was not calibrated.");
            }
        }
        else
        {
            throw new System.Exception("The segment for " + anatPoint.name + " is not
visible. The anatomical point was not calibrated.");
        }
    }
    else
        return false;
}

private void UpdateLocalCoordinates(Marker anatPoint, Segment parentSegment, Marker
calibrationWand)
{
    Vector<double> P_glob = calibrationWand.globalCoordinates -
parentSegment.position;
    Matrix<double> R = RotMatrices.invZXZ(parentSegment.orientation);
    Vector<double> P_loc = R * (P_glob);

    anatPoint.localCoordinates = P_loc;
}
```

Calculation of the calibrated anatomical point positions through homogeneous coordinate transformation is continuously performed after the anatomical calibration with the following function.

```
private void UpdateAnatPointCoordiantes(Marker AnatPoint, Segment parentSegment)
{
    Matrix<double> R = RotMatrices.ZXZ(parentSegment.orientation);

    Matrix<double> T = DenseMatrix.OfArray(new double[,] {
                {R[0,0], R[0,1] , R[0,2] ,parentSegment.position[0]},
                {R[1,0], R[1,1] , R[1,2] ,parentSegment.position[1]},
                {R[2,0], R[2,1] , R[2,2] ,parentSegment.position[2]},
                {0,0,0,1}});

    Vector<double> P_loc = Vector<double>.Build.Dense(4);
    P_loc[0] = AnatPoint.localCoordinates[0];
    P_loc[1] = AnatPoint.localCoordinates[1];
    P_loc[2] = AnatPoint.localCoordinates[2];
    P_loc[3] = 1;

    Vector<double> P_glob = T * P_loc;

    AnatPoint.globalCoordinates[0] = P_glob[0];
    AnatPoint.globalCoordinates[1] = P_glob[1];
    AnatPoint.globalCoordinates[2] = P_glob[2];

    AnatPoint.isVisible = true;
}
```
