# Supplementary figures and images for: Affordable gait analysis using augmented reality markers
Source: PLoS One. 2019 Feb 14;14(2):e0212319. doi: 10.1371/journal.pone.0212319 (PMC6375625; doi:10.1371/journal.pone.0212319)

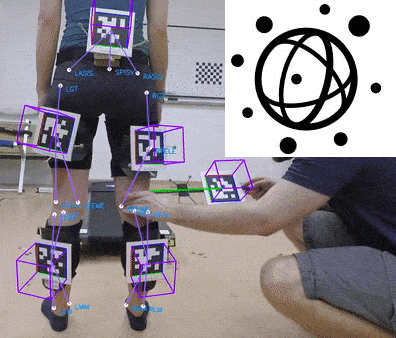

Supplement: S1 Fig — (GIF) [file pone.0212319.s006.gif]

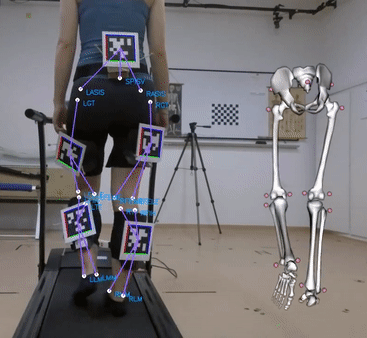

Supplement: S2 Fig — (GIF) [file pone.0212319.s007.gif]
